# Effect of hydrocolloids and emulsifiers on the shelf‐life of composite cassava‐maize‐wheat bread after storage

**DOI:** 10.1002/fsn3.326

**Published:** 2016-02-28

**Authors:** Maria Eduardo, Ulf Svanberg, Lilia Ahrné

**Affiliations:** ^1^ Departamento de Engenharia QuímicaFaculdade de EngenhariaUniversidade Eduardo MondlaneMaputoMoçambique; ^2^ Department of Biology and Biological Engineering/Food and Nutrition ScienceChalmers University of TechnologyGothenburgSweden; ^3^ Process and Technology developmentSP Technical Research Institute of SwedenFood and BioscienceGothenburgSweden

**Keywords:** Baking improvers, bread quality, Cassava flour, starch retrogradation, storage

## Abstract

The objective of this study was to evaluate the effect of hydrocolloids and/or emulsifiers on the shelf‐life of composite cassava‐maize‐wheat (ratio 40:10:50) reference bread during storage. Added hydrocolloids were carboxymethylcellulose (CMC) and high methoxyl pectin (HM pectin) at a 3% level (w/w) and/or the emulsifiers diacetyl tartaric acid esters of monoglycerides (DATEM), lecithin (LC), and monoglycerides (MG) at a 0.3% level (w/w). After 4 days of storage, composite breads with MG had comparatively lower crumb moisture while crumb density was similar in all breads. The reference bread crumb firmness was 33.4 N, which was reduced with an addition of DATEM (23.0 N), MG (29.8 N), CMC (24.6 N) or HM pectin (22.4 N). However, the CMC/DATEM, CMC/LC, and HM pectin/DATEM combinations further reduced crumb firmness to <20.0 N. The melting peak temperature was increased from 52 C to between 53.0 C and 57.0 C with added hydrocolloids and/or emulsifiers. The melting enthalpy of the retrograded amylopectin was lower in composite bread with hydrocolloids and emulsifiers, 6.7–11.0 J/g compared to 20.0 J/g for the reference bread. These results show that emulsifiers in combination with hydrocolloids can improve the quality and extend the shelf‐life of composite cassava‐maize‐wheat breads.

## Introduction

Partial replacement of wheat flour by flours produced from locally grown crops such as cassava and maize has a major economic interest to reduce the dependence on expensive wheat imports. The challenge in substituting wheat flour lies in the fact that the bread quality is mainly governed by the gluten content of wheat, which becomes gradually lower with increasing amounts of alternative flours (>20%), leading to poor ability of the gluten proteins to form a cohesive and viscoelastic dough during baking and retain the gas formed during the fermentation. Consequently, the bread produced has a lower volume and a compact crumb structure.

To compensate for the poor gluten network Eduardo et al. (2014a) studied the effect of hydrocolloids and emulsifiers on improvement of the quality of composite bread evaluated as specific loaf volume, crumb moisture and firmness, and crust color. Addition of hydrocolloids (3% w/w), carboxymethylcellulose (CMC), or high methoxyl pectin (HM pectin), combined with different types of emulsifiers (0.1–0.5% w/w), diacetyl tartaric acid ester of monoglycerides (DATEM), sodium stearoyl lactylate (SSL) or lecithin (LC) showed that the specific loaf volume and bread firmness of the composite bread were significantly improved by the combination of hydrocolloids and emulsifiers. Based on the results of this study, two optimized bread formulations consisting of either CMC/DATEM or HM pectin/LC, both at ratio of 3:0.3%, were selected for sensorial and consumer studies in Mozambique. The results showed a high acceptability and willingness to purchase composite bread based on cassava flour among Mozambican consumers (Eduardo et al. 2014b).

Replacement of wheat by starch rich flours like cassava is also expected to affect the shelf‐life of bread due to the increased amount of starch which can undergo retrogradation during storage and cause bread firmness to increase and consequently results in a loss of quality. Retrogradation of starch includes the short‐term development of a gel network structure of amylose (crystallization) and a long‐term reordering of amylopectin, which is a much slower process involving recrystallization of the outer branches of this polymer (Miles et al. 1985; Ring et al. 1987). Retrogradation of starch is also affected by the redistribution of water between starch and gluten and, as a result, the crumb will become increasingly firm with time (Eliasson and Larsson 1993; Davidou et al. 1996; Purhagen et al. 2012). According to Gray and Bemiller (2003), amylopectin is the major factor in the retrogradation process but is not solely responsible for the observed change in texture. However, the mechanisms for these processes are still not completely understood.

The addition of hydrocolloids and emulsifiers to cassava composite bread are expected to influence the retrogradation of starch and consequently the shelf‐life of bread. Hydrocolloids can increase water retention capacity influencing the water redistribution and consequently the retrogradation. Davidou et al. (1996) reported a decrease firmness and starch retrogradation during storage in wheat bread by addition of locust bean gum, alginate, and xanthan.

Emulsifiers are commonly used in bakery products to improve softness of the crumb (Demirkesen et al. 2010). They are composed of both hydrophobic and hydrophilic residues, which allow the interaction and formation of complexes with starch, protein, shortening, and water. The improving effect of emulsifiers seems to be related to their effect in reducing the repulsing charges between gluten proteins by causing them to aggregate in composite dough flour as the wheat gluten has been diluted. For instance, interaction of an emulsifier with the protein can improve dough strength and allow better retention of carbon dioxide (Demirkesen et al. 2010).

The combination of hydrocolloids and emulsifiers might have synergistic effects leading to longer shelf‐life, but no studies on composite bread have been found in literature. The objective of this study was therefore to investigate the effect of hydrocolloids (HM pectin and CMC) and emulsifiers (DATEM, LC, and MG) and their combined effect on extension of shelf‐life of optimized composite cassava‐maize‐wheat bread up to 4 days of storage.

## Materials and Methods

### Materials

Wheat flour of 10.5% protein (Bagerivetemjöl, Frebaco Kvarn, Sweden), yellow maize flour of 7.14% ± 0.05 protein)(AB Risenta, Sweden), roasted cassava flour of 1.35% ± 0.07 protein (kjeldahl method N × 6.25) (AOAC, 1990), instant dry yeast, salt, sugar, vegetable oil, and ascorbic acid (Merck Chemicals, Germany) were used for bread making.

Hydrocolloids were HM pectin (degree of esterification 68–75%) (GENU pectin type BIG, CP Kelco, Denmark), CMC (degree of substitution 0.75–0.85) (CEKOL 50000 W, CP Kelco, Finland), and emulsifiers were DATE M (MULTEC HP 20, Puratos, Belgium), soy lecithin, LC (phosphatidylcholine min. 18%, phosphatidylinositol min. 13%, and phosphatidylethanolamine min. 15%) (LECICO P 900 IPM, Lecico GmbH, Hamburg, Germany), and monoglyceride, MG (total monoglyceride min. 90%, free glycerol max. 1% and acid value max. 3 mg KOH/g) (Dimodan® PH200, DANISCO, Denmark). The emulsifiers were selected according to their difference in hydrophilic lipophilic balance values (HLB). DATEM (HLB value of 9.2) and lecithin (HLB value between 3 and 4) are both anionic oil‐in‐water emulsifiers, which might influence protein denaturation (Pisesookbunterng and D'Appolonia 1983). Monoglyceride (HLB value between 2.8 and 3.8) is commonly used in bakeries to delay staling. This is a nonionic water‐in‐oil emulsifier. Fresh cassava roots were obtained from local producers in Mozambique and processed into roasted cassava flour as previously described (Eduardo et al. 2013) and was as follows. The roots (~100 kg) was peeled, washed in potable water and manual chipped, which was moist fermented for about 2 days. The fermented cassava was pressed, screened with a mechanical machine, and toasted in a frying pan until cooked and crisp (~10 min). The toasted material was milled in a laboratory mill with a sieve DIN 4188 (0.125 mm aperture sieve). The flour (~20 kg) was then packed in polyethylene bags until use.

### Test baking

The test baking experiments were randomized. Composite breads were produced from cassava‐maize‐wheat flours that contained either HM pectin or CMC at a level of 3% on a flour weight basis. Subsequent tests involved the combination of either 0.3% of DATEM, LC or MG with each of the above levels of HM pectin and CMC. A model system prepared with each different emulsifier was also examined. A control loaf was produced that contained no improvers. The recipe is given in Table 1.

**Table 1:** Bread formulation

Ingredients	%
Flour (50% wheat, 40% cassava and 10% maize)	100.0
Dry yeast	1.6
Salt	1.5
Sugar	2.0
Oil	3.0
Ascorbic acid	0.1
Hydrocolloids (CMC or HM pectin)	3.0
Emulsifiers (DATEM, LC, and MG)	0.3
Water (at 15.5°C)	88.3

The dough was mixed in a KSM9 mixer (KitchenAid, USA) for two minutes at low speed followed by eight minutes of mixing at medium speed. The dough (1500 g) was covered with a kitchen cloth and allowed to rest at room temperature for 45 min. At the end of the resting period, the dough was divided (into pieces of 50 g), molded by hand and placed in aluminum pans. The loaves were proofed for 45 min at 30°C and 80% relative humidity (RH) in a fermentation cabinet (Labrum Klimat Ab, Stockholm, Sweden) and baked at 220°C for 7 min in a rotating convention oven (Dahlen S400, Sveba Dahlen AB, Sweden) with air circulation. Before measurements, the breads were cooled for 1 h at room temperature. Afterward, the unpacked bread loaves were stored in a room with controlled relative humidity (50%) and temperature (23°C) for 4 days until further characterization.

The composite breads were analyzed for weight, crumb moisture, density, and melting enthalpy of the retrograded amylopectin on the baking (day 0) and 4 days later. Firmness was measured after 3 h and 1, 2, 3, and 4 days of storage.

### Analysis of composite bread

#### Weight and volume

The weight of the loaves (*n* = 6) was measured after cooling on day 0 and day four. The volume (*n* = 6) was measured using the rapeseed displacement method, where alfalfa seed was used instead of millet. Each loaf was weighed, and the specific loaf volume (cm^3^/g) was calculated as loaf volume (cm^3^)/loaf weight (g) taken after 1 h of baking.

#### Crumb density

The density (*ρ*) (g/cm^3^) corresponding to the density of the material of the cell walls (*n* = 2) was determined with a gas pycnometer (AccuPyc II 1340, CIAB, Sweden) using nitrogen as the displacement fluid.

#### Crust color

The instrumental measurement of the bread crust color (*n* = 4) was carried out with Digital Colour Imaging System (DigiEye) (Cromocol Scandinavia AB, Borås, Sweden). The controlled illumination cabinet on the DigiEye equipment was utilized to capture high‐resolution images of the fresh bread surface. The DigiEye 2.53b software (Cromocol Scandinavia AB, Borås, Sweden) allows for storage of specific color standards with given L* (lightness), a* (redness‐greenness), and b* (yellowness‐blueness) values according to the CIELab system definition. The results were reported as the browning index (BI) of the bread crust as calculated by Maskan (2001).

#### Crumb moisture

The crumb moisture (g of water/100 g, wet sample) (*n* = 3) was determined in triplicate by drying the samples overnight under a vacuum oven at 70°C under 29 in. of Hg (AACC method 44–40, 1995).

#### Crumb texture

The texture properties of the crumb were measured using an Instron 5542 universal testing machine (Canton, MA, USA). A modified AACC standard method 74–09 was used with a cylindrical probe (diameter 15 mm). The crumb samples (*n* = 4) of composite bread (2.5 cm) were compressed to 40% at a crosshead speed of 1.7 mm/sec. Firmness is the parameter that describes the resistance to compression of the bread crumbs.

#### Thermal properties

Analyses were performed in a DSC‐1 (Mettler Toledo AB, Sweden) using a medium pressure pan. The equipment was calibrated with Indium (enthalpy of fusion 28.41 J/g, melting point 156.4°C), and an empty pan was used as a reference. Approximately 90 mg of crumb:water (ratio 1:2 w/w) was weighed in the pan, which was hermetically sealed in order to avoid moisture loss. Samples (*n* = 3) were heated from 20 to 130°C with a 5°C/min scanning rate (Sahlström et al. 2003). The onset temperature (T_o_), the peak temperature (T_p_), and the transition enthalpy (J/g, dry sample) of amylopectin crystals (retrogradation) (∆H_retro_) were evaluated from the thermograms using the program Mettler Star^e^ (Mettler‐Toledo GMbH, Schwerzenbach, Switzerland).

### Statistical analysis

SPSS software version 16.0 (SPSS Inc., Chicago, IL, USA) was used to analyze the data obtained. One‐way ANOVA was used in data from the composite bread analysis. Tukey HSD (honesty significant difference) post hoc mean comparison tests were used to detect significant differences at a confidence level of 95% (*P < 0.05*). The mean values tested were calculated based on at least two to six individual measurements of one batch of bread.

## Results and Discussion

### Effect of hydrocolloids and emulsifiers on the characteristics of composite breads

Adding either hydrocolloids or emulsifiers or combinations of hydrocolloid/emulsifier to composite dough formulation significantly increased the specific volume compared with the reference bread (with no improver) (Table 2). The largest increase in specific volume was obtained with a combination of CMC/MG (28%), followed by CMC/DATEM (21%) and CMC/LC (19%). However, the specific volume of the loaves with either emulsifiers or hydrocolloids was not significantly different. These results are in agreement with Rosell et al. (2001), Guarda et al. (2004), Bárcenas and Rosell (2005) and Correa et al. (2012), who found an increased loaf volume of wheat bread with an addition of the hydrocolloids HPMC (hydroxypropyl methylcellulose), HM pectin and *κ*‐carrageenan, and similar findings have been reported in gluten‐free breads with additions of DATEM (Nunes et al. 2009; Demirkesen et al. 2010), distilled monoglyceride (MG), and lecithin (Nunes et al. 2009). However, no effect was observed with an addition of MG and DATEM in gluten‐free bread formulations with mixtures of rice flour and cassava starch (Sciarini et al. 2012). The positive effect of hydrocolloids and/or emulsifiers on the volume of the bread is explained by an increased stability of the dough system during proofing (hydrocolloids) (Guarda et al. 2004) and by the formation of a stabilized liquid film lamellae/gas cell interface (emulsifiers) (Selmair and Koehler 2010). As a result, additional strength was conferred to the gas cells of the dough, thereby increasing gas retention and/or oven spring. This led as a consequence to higher bread volume (Gómez et al. 2004; Guarda et al. 2004).

**Table 2:** Specific volume and brownness index of fresh composite bread samples as affected by hydrocolloids, emulsifiers, and combinations of both improvers

Bread formulations	Specific volume (cm^3^/g)	Brownness index (color units)
No emulsifier or hydrocolloid	1.93 ± 0.06^a^	37.9 ± 2.1^a^
Emulsifiers (0.3%)*:*		
DATEM	2.08 ± 0.10^b^	47.6 ± 1.2^b^
LC	2.07 ± 0.06^b^	44.1 ± 1.9^ab^
MG	2.07 ± 0.05^b^	43.2 ± 1.8^ab^
Hydrocolloids (3%):		
CMC	2.10 ± 0.07^b^	43.6 ± 3.8^ab^
HM pectin	2.12 ± 0.04^bc^	56.1 ± 3.3^cd^
Hydrocolloids (3%) + Emulsifiers (0.3%):		
CMC/DATEM	2.34 ± 0.06^d^	63.4 ± 2.4^e^
HM pectin/DATEM	2.11 ± 0.01^b^	49.9 ± 1.9^bc^
CMC/LC	2.30 ± 0.05^d^	55.1 ± 3.4^cd^
HM pectin/LC	2.23 ± 0.02^cd^	58.4 ± 1.0^de^
CMC/MG	2.46 ± 0.11^e^	60.7 ± 5.6^de^
HM pectin/MG	2.09 ± 0.06^b^	44.3 ± 3.6^ab^

Values in the same column followed by different letters are significantly different (*P *< 0.05).

CMC, carboxymethyl cellulose; HM pectin, high methoxyl pectin; DATEM, diacetyl tartaric acid esters of monoglycerides; LC, lecithin; MG, monoglycerides.

Hydrocolloids and emulsifiers or combinations of them also affected the crust color, that is, the brownness index (BI) of the composite cassava‐maize‐wheat breads. The BI was higher (≥58) in the bread crust of CMC combined with DATEM or MG and HM pectin with LC; BI values above of 58 can be considered to be preferred by consumers (Eduardo et al. 2014b). The increased BI value could be attributable to a more favorable water distribution due to the hydrocolloids, which affects Maillard browning reactions and caramelization (Sciarini et al. 2010).

### Effect of the hydrocolloids and emulsifiers on the characteristics of stored composite breads

#### Weight

The weight of fresh loaves of bread (with or without improvers) ranged between 44.5 g and 45.7 g (Table 3), whereas the weight of the loaf of the reference bread was 45.4 g. The weight of the bread was in general slightly lower in composite breads with improvers, which means that the weight loss (approx. 10% w/w) during the baking process was in general 1% w/w higher in breads with improvers.

**Table 3:** Weight, crumb density, crumb moisture, and crumb firmness of fresh and stored composite bread samples as affected by hydrocolloids, emulsifiers, and combinations of both improvers

	Weight (g)		Crumb density (g/cm^3^)		Crumb moisture (% wet basis)		Crumb firmness (N)	
Bread formulations	Fresh bread	4‐d storage	Fresh bread	4‐d storage	Fresh bread	4‐d storage	Fresh bread	4‐d storage
No emulsifier or hydrocolloid	45.4 ± 0.2^cd^	32.2 ± 0.3^b^	1.30 ± 0.01^ab^	1.38 ± 0.00^a^	48.6 ± 0.2^abc^	27.7 ± 0.2^c^	6.9 ± 0.2^f^	33.4 ± 0.6^g^
Emulsifiers (0.3%):								
DATEM	44.9 ± 0.2^ab^	32.4 ± 0.3^b^	1.31 ± 0.02^ab^	1.36 ± 0.00^a^	48.8 ± 0.2^bc^	29.3 ± 0.1^cd^	5.0 ± 0.3^d^	23.0 ± 0.3^d^
LC	45.4 ± 0.3^bcd^	33.5 ± 0.6^cd^	1.29 ± 0.01^ab^	1.35 ± 0.00^a^	47.8 ± 0.1^ab^	28.8 ± 1.1^cd^	6.0 ± 0.5^e^	36.5 ± 0.6^h^
MG	44.8 ± 0.2^a^	30.8 ± 0.2^a^	1.28 ± 0.02^ab^	1.39 ± 0.03^a^	48.6 ± 0.6^abc^	24.8 ± 0.9^a^	5.7 ± 0.2^e^	29.8 ± 0.4^f^
Hydrocolloids (3%):								
CMC	45.7 ± 0.3^d^	33.9 ± 0.5^d^	1.29 ± 0.01^ab^	1.36 ± 0.01^a^	47.5 ± 0.8^a^	29.5 ± 0.3^cd^	4.2 ± 0.2^bc^	24.6 ± 0.4^e^
HM pectin	44.8 ± 0.1^a^	32.2 ± 0.3^b^	1.31 ± 0.00^ab^	1.38 ± 0.01^a^	48.0 ± 0.3^ab^	27.5 ± 1.5^bc^	3.8 ± 0.1^abc^	22.3 ± 0.5^d^
Hydrocolloids (3%) + Emulsifiers (0.3%):								
CMC/DATEM	44.9 ± 0.3^abc^	32.5 ± 0.5^b^	1.30 ± 0.00^ab^	1.34 ± 0.00^a^	49.7 ± 0.2^c^	31.2 ± 1.3^d^	3.6 ± 0.1^a^	12.4 ± 0.4^a^
HM pectin/DATEM	45.0 ± 0.3^abc^	32.8 ± 0.3^bc^	1.33 ± 0.00^b^	1.38 ± 0.01^a^	48.2 ± 0.5^ab^	29.3 ± 1.0^cd^	4.0 ± 0.1^abc^	17.0 ± 0.5^b^
CMC/LC	45.0 ± 0.1^abc^	32.3 ± 0.2^b^	1.28 ± 0.02^ab^	1.37 ± 0.00^a^	47.8 ± 0.4^ab^	27.3 ± 0.5^bc^	3.8 ± 0.2^abc^	16.6 ± 0.5^b^
HM pectin/LC	44.9 ± 0.3^ab^	32.8 ± 0.3^bc^	1.30 ± 0.02^ab^	1.38 ± 0.02^a^	48.1 ± 0.6^ab^	29.0 ± 0.5^cd^	3.7 ± 0.1^ab^	20.1 ± 0.6^c^
CMC/MG	44.5 ± 0.3^a^	31.3 ± 0.4^a^	1.27 ± 0.01^a^	1.36 ± 0.00^a^	47.9 ± 0.2^ab^	25.3 ± 0.9^ab^	4.3 ± 0.1^c^	21.8 ± 0.4^d^
HM pectin/MG	44.9 ± 0.2^abc^	31.3 ± 0.2^a^	1.27 ± 0.01^a^	1.38 ± 0.05^a^	47.7 ± 0.4^ab^	24.7 ± 0.7^a^	5.7 ± 0.1^e^	29.2 ± 0.6^f^

Values in the same column followed by different letters are significantly different (*P < 0.05*).

CMC, carboxymethyl cellulose; HM pectin, high methoxyl pectin; DATEM, diacetyl tartaric acid esters of monoglycerides; LC, lecithin; MG, monoglycerides.

Over 4 days of storage, a significant reduction in weight was observed for all bread samples as a result of moisture loss. However, as compared with the reference bread, a significantly higher bread weight was obtained in loaves containing CMC and LC (about 4%), and significantly lower weights for all loaves with MG (about 3%). All the other bread loaves had a weight similar to that of the reference bread, which might indicate that the addition of improvers had little effect on water loss during 4 days of storage. This may be explained by the very high water binding capacity of pregelatinized starch (Seyhun et al. 2005), since the cassava flour used in this study was partially pregelatinized; hydrocolloids have otherwise been shown to reduce the crumb dehydration rate during storage (Guarda et al. 2004).

#### Crumb density

The density of the fresh composite bread crumb was 1.30 g/cm^3^. In general, no significant differences were found between the reference bread and breads baked with hydrocolloids, emulsifiers, or their combinations. The crumb density of all the composite breads with baking improvers increased after 4 days in relation to the initial value (the baking day) and was in the range of 1.34 g/cm^3^ and 1.39 g/cm^3^, similar to the reference bread (1.38 g/cm^3^). Lagrain et al. (2012) reported that bread density is a major determining factor of bread crumb structure and texture during storage. In breads with a similar density and crumb structure, the evolution of crumb stiffness during storage was determined by changes in the starch component.

#### Crumb moisture

The crumb moisture content of the fresh breads varied between 47.5% and 49.7%. None of the tested hydrocolloids, emulsifiers and their combinations affected the bread moisture content significantly due to the fact that all bread samples were produced using approximately the same amount of water (≈ 88% of flour weight).

However, the moisture content decreased in all bread samples stored for 4 days in comparison with the initial value (Table 3), and a significantly lower moisture content was found for all breads with MG as compared to the reference bread. In contrast, bread with DATEM/CMC had a higher moisture content. A higher moisture content in the crumb is preferred for better quality during storage, as it will give softer crumbs with less crumb hardening (Guarda et al. 2004). The decrease in crumb moisture is known to affect the crumb firming rates (He and Hoseney 1990) by the formation of cross links between partially solubilized starch and gluten proteins (Martin et al. 1991).

#### Firmness

**Figure 1:**
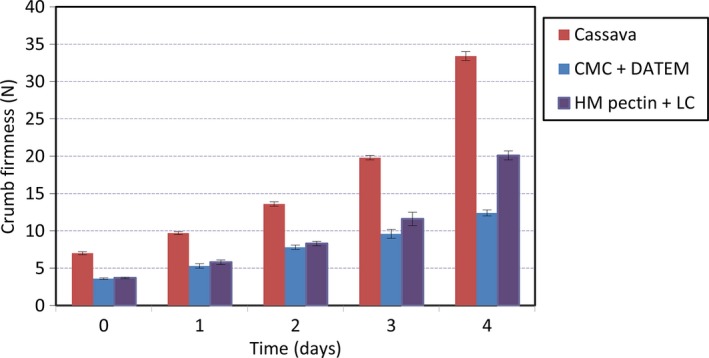
Effects of hydrocolloids (CMC and HM pectin) and their interactive effect with emulsifiers (DATEM and LC) on the crumb firmness of composite cassava‐maize‐wheat breads during storage (23°C and 50% r.h.). Error bars indicate the standard deviation. Lc*,* lecithin.

The firmness of the fresh reference composite bread crumb was 6.9 N, whereas all breads with baking improvers had a significantly lower crumb firmness, between 3.7 and 6.0 N (Table 3). All composite bread samples increased in hardness during storage in relation to the initial value (the first day). However, composite breads baked with baking improvers had a crumb firmness after 2 days of storage similar to fresh reference bread with no improvers (Fig. 1). After 4 days of storage, the crumb firmness of the reference bread increased to 33.4 N as a result of the staling process and loss of moisture. Breads with improvers (except bread baked with LC alone, Table 3) maintained a significantly softer crumb in comparison with the reference bread after 4 days of storage. Our results that hydrocolloids have a crumb softening effect on both fresh and stored composite bread is in agreement with Guarda et al. (2004) and Correa et al. (2012), who found that HPMC and pectin decreased the crumb hardness in a wheat bread. The effect was explained by a decreased loss of water during storage (high water retention capacity of the hydrocolloids), and crumb hardening was retarded as a result. This effect could be related to the inhibition of the amylopectin retrogradation due to the water retention capacity of hydrocolloids, that is, recrystallization of amylopectin is retarded at lower water availability (Zeleznak and Hoseney 1986; Guarda et al. 2004). However, our results differ from those obtained by Lazaridou et al. (2007), who reported that pectin and CMC did not affect the crumb firmness of gluten‐free breads. The contrasting results can probably be explained by the different bread formulations (gluten‐free and composite cassava‐maize‐wheat).

The only bread with a firmness higher than the reference bread was the bread with LC. Some studies indicate that LC only has a small delaying effect on the firming of wheat starch bread (Forssell et al. 1998), or no delaying effect on the crumb firming of wheat bread (Stampfli and Nersten 1995) or gluten‐free bread (Nunes et al. 2009). The increase in bread firmness with LC was explained by its inability to form complexes with the starch (Stampfli and Nersten 1995; Forssell et al. 1998).

With respect to the antistaling effect of emulsifiers, Pisesookbunterng and D'Appolonia (1983) suggested that the adsorption of emulsifier to the starch granule, as well as the formation of a starch‐emulsifier complex, restrained the starch from taking up water released from gluten during the aging of the bread. Moreover, monoglycerides, which form strong complexes with amylose, will reduce granule swelling and solubilization (Gray and Schoch 1962; Gómez et al. 2004). The reduction in starch swelling and the degree of granule swelling are inversely related to crumb firmness. Lecithin, with a higher content of lysophospholipids, has been reported to retard bread staling (Forssell et al. 1998; Gray and Bemiller 2003) by complexing with starch amylose (Forssell et al. 1998). DATEM, however, initially produces lower crumb firmness and then retards the rate of staling through its interaction with not only the amylose but also with amylopectin (Kamel and Ponte 1993; Gray and Bemiller 2003).

The combined effect of emulsifiers and hydrocolloids in reducing bread hardness was thus more pronounced than when either was added separately.

### Retrogradation of starch

The amylose is responsible for setting the initial network structure but is not involved in long‐term staling (Eliasson and Larsson 1993). The long‐term change after 4 days of storage is therefore attributed to the amylopectin fraction, which in our composite bread is assumed to constitute about 75% of the total starch.

After storage for 4 days, an endothermal staling peak appears on the DSC thermogram in the range of 35–70°C (Schiraldi et al. 1996) as a result of the melting enthalpy of recrystallized amylopectin (ΔH_**retro**_). The hydrocolloids, emulsifiers and the combinations of both exhibited different results in their effect on starch retrogradation after 4 days of storage (Table 4). However, in fresh bread no retrogradation peak was observed as previously observed by Purhagen et al., (2008).

**Table 4:** Thermal properties of composite cassava‐maize‐wheat bread stored for 4 days as affected by hydrocolloids, emulsifiers, and a combination of both improvers

Composite bread samples	T_o_ (°C)	T_p_ (°C)	∆H_retro_ (J/g dry crumb)
No emulsifier or hydrocolloid	51.1 ± 0.4	52.1 ± 0.8	20.0 ± 0.2^g^
Emulsifiers (0.3%):			
DATEM	51.2 ± 1.2	54.7 ± 1.4	14.0 ± 0.2^def^
LC	51.9 ± 1.0	54.7 ± 2.1	23.2 ± 0.4^h^
MG	52.1 ± 0.6	52.9 ± 0.6	15.6 ± 0.8^f^
Hydrocolloids (3%):			
CMC	51.5 ± 2.2	54.8 ± 0.6	12.8 ± 0.5^cd^
HM pectin	51.6 ± 0.3	56.4 ± 1.6	14.8 ± 0.8^ef^
Hydrocolloids (3%) + emulsifiers (0.3%):			
CMC/DATEM	50.6 ± 0.7	55.2 ± 1.5	10.6 ± 0.3^b^
HM pectin/DATEM	52.8 ± 0.5	55.6 ± 1.1	10.3 ± 0.1^b^
CMC/LC	52.8 ± 1.4	55.2 ± 2.6	6.7 ± 0.4^a^
HM pectin/LC	52.9 ± 2.0	55.6 ± 1.3	12.8 ± 0.6^cde^
CMC/MG	50.8 ± 0.7	55.1 ± 0.5	11.1 ± 0.9^bc^
HM pectin/MG	53.4 ± 1.5	56.9 ± 1.4	15.8 ± 0.6^f^

Values in the fourth column followed by different letters are significantly different (*P < 0.05*).

T_o_, onset temperature; T_p_, peak temperature; ∆H_retro_, enthalpy of melting of the amylopectin recrystallization; CMC, carboxymethyl cellulose; HM pectin, high methoxyl pectin; DATEM, diacetyl tartaric acid esters of monoglycerides; LC, lecithin; MG, monoglycerides.

As Table 4 shows, the onset temperature (T_o_) and melting enthalpy (∆H_retro_) of recrystallized amylopectin of composite bread varied from 50.6–53.4°C and 6.7–23.2 J/g dry crumb, respectively, depending on the hydrocolloids (CMC and HM pectin) and/or the emulsifier types (DATEM, LC, and MG). In the reference composite bread, the retrogradation peak temperature appeared at 52°C. The addition of hydrocolloids and/or emulsifiers had a peak temperature that was 1.9–4.8°C above that of the reference bread, which indicates that the melting of recrystallized amylopectin enthalpy was delayed. The addition of improvers, except LC, in the composite bread loaves significantly reduced the melting enthalpy values compared to that of the reference bread (20.0 J/g). The lowest value was observed for CMC/LC (6.7 J/g), followed by HM pectin/DATEM (10.3 J/g) and CMC/DATEM (10.6 J/g), whereas LC showed the highest value (23.2 J/g).

Schiraldi et al. (1996) and Gujral et al. (2004) also found decreased starch retrogradation with hydrocolloids, which confirms the findings obtained in this study. The effect of hydrocolloids on starch retrogradation seems to be due to their interaction with water by limiting moisture transfer and loss and also with starch chains in the mixture (Davidou et al. 1996; Gavilighi et al. 2006). Purhagen et al. (2012) also observed that an addition of DATEM gave less retrograded amylopectin in gluten‐free bread, which was attributed to the formation of amylose‐emulsifier complex, thereby preventing amylopectin from re‐crystallizing (retrograde) (Gudmundsson and Eliasson 1990). The effect of the addition of both hydrocolloids and emulsifiers results from an increase in the starchy lipids due to preferential binding to the gluten and displacement of nonstarchy bound lipids to the starch, and an increase in the free lipids, respectively, thus lowering the firming rate of the bread (Collar et al. 2001).

**Figure 2:**
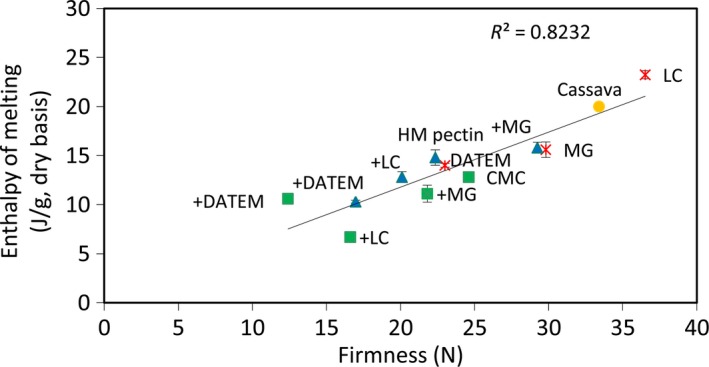
Relationship between amylopectin recrystallization, measured as enthalpy of melting, and firmness of composite cassava‐maize‐wheat bread crumb samples with hydrocolloids (CMC and HM pectin), emulsifiers (DATEM, lecithin (LC), and MG) and a combination of both improvers stored at 23°C and 50% for 4 days of storage.

Figure 2 shows the enthalpy of melting recrystallized amylopectin as a function of the firmness of composite cassava‐maize‐wheat bread crumb samples after 4 days of storage. In general, the firmness of the bread crumbs increased with increased enthalpy of melting for all composite bread samples (with or without improvers) (*R* ^*2*^ = 0.82). Bread baked with LC had a significantly higher firmness and enthalpy of melting values compared with the reference bread (Tables 3 and 4). However, the composite bread prepared with the combination of CMC/DATEM had the lowest crumb firmness (*P* < 0.05), which corresponds to the lowest enthalpy of melting recrystallized amylopectin.

In general, composite bread with CMC had a lower enthalpy of melting recrystallized amylopectin and firmness compared with the corresponding bread with HM pectin. The explanation may be that HM pectin more strongly binds the water content in the bread and thereby gives rise to a more rapid firming rate (Rogers et al. 1988). However, the retrogradation rate has been shown to be lower at low moisture starch gels (Zeleznak and Hoseney 1986).

The different types of improvers added to composite dough formulations seems to influence the rate of amylopectin recrystallization and consequently retards starch retrogradation in composite cassava‐maize‐wheat breads.

## Conclusions

This study has shown that an addition of hydrocolloids (CMC and HM pectin) and/or emulsifiers (DATEM, LC, and MG) to composite cassava‐maize‐wheat bread has varying effects on quality parameters of breads during storage.

After 4 days of storage, the density and firmness of the stored bread loaves increased while the weight and moisture content was reduced in comparison with the fresh bread. Addition of emulsifiers (DATEM and MG) reduced crumb firmness but did not show a significant effect on the weight, density, or crumb moisture compared to the reference bread (with no improver). The main effect of the hydrocolloids was reduced crumb firmness, and the combination of DATEM with CMC showed the lowest crumb firmness after storage.

We found that the hydrocolloids and emulsifiers delayed the melting peak temperature for retrogradation and that the combination of both improvers further reduced the retrogradation peak temperature. CMC/LC, HM pectin/DATEM, and CMC/DATEM were especially effective in retarding starch recrystallization in composite cassava‐maize‐wheat bread. This suggests that emulsifiers in combination with hydrocolloids have a significant effect on retarding starch retrogradation in composite cassava‐maize‐wheat bread.

## Conflict of Interest

None declared.
